# Sperm Associated Antigen 9 Plays an Important Role in Bladder Transitional Cell Carcinoma

**DOI:** 10.1371/journal.pone.0081348

**Published:** 2013-12-09

**Authors:** Deepika Kanojia, Manoj Garg, Shikha Saini, Sumit Agarwal, Deepak Parashar, Nirmala Jagadish, Amlesh Seth, Amar Bhatnagar, Anju Gupta, Rajive Kumar, Nirmal Kumar Lohiya, Anil Suri

**Affiliations:** 1 Cancer Microarray, Genes and Proteins Laboratory, National Institute of Immunology, Aruna Asaf Ali Marg, New Delhi, India; 2 Department of Urology, All India Institute of Medical Sciences, Ansari Nagar, New Delhi, India; 3 Department of Cancer Surgery, Safdarjung Hospital and Vardhman Mahavir Medical College, New Delhi, India; 4 NMC Imaging and Diagnostic Centre, Vidyasagar Institute of Mental Health and Neuro-Sciences, New Delhi, India; 5 Institute of Rotary Cancer Hospital, All India Institute of Medical Sciences, New Delhi, India; 6 Reproductive Physiology Section, Department of Zoology, University of Rajasthan, Jaipur, India; Taipei Medicine University, Taiwan

## Abstract

**Background:**

Majority of bladder cancer deaths are caused due to transitional cell carcinoma (TCC) which is the most prevalent and chemoresistant malignancy of urinary bladder. Therefore, we analyzed the role of Sperm associated antigen 9 (SPAG9) in bladder TCC.

**Methodology and Findings:**

We examined SPAG9 expression and humoral response in 125 bladder TCC patients. Four bladder cancer cell lines were assessed for SPAG9 expression. In addition, we investigated the effect of SPAG9 ablation on cellular proliferation, cell cycle, migration and invasion in UM-UC-3 bladder cancer cells by employing gene silencing approach. Our SPAG9 gene and protein expression analysis revealed SPAG9 expression in 81% of bladder TCC tissue specimens. High SPAG9 expression (>60% SPAG9 positive cells) was found to be significantly associated with superficial non-muscle invasive stage (*P = *0.042) and low grade tumors (*P = *0.002) suggesting SPAG9 putative role in early spread and tumorigenesis. Humoral response against SPAG9 was observed in 95% of patients found positive for SPAG9 expression. All four bladder cancer cell lines revealed SPAG9 expression. In addition, SPAG9 gene silencing in UM-UC-3 cells resulted in induction of G_0_–G_1_ arrest characterized by up-regulation of p16 and p21 and consequent down-regulation of cyclin E, cyclin D and cyclin B, CDK4 and CDK1. Further, SPAG9 gene silencing also resulted in reduction in cellular growth, and migration and invasion ability of cancer cells *in vitro*.

**Conclusions:**

Collectively, our data in clinical specimens indicated that SPAG9 is potential biomarker and therapeutic target for bladder TCC.

## Introduction

Bladder transitional cell carcinoma (TCC) is one of the tumors associated with the highest morbidity and mortality among genitourinary malignancies, accounting for 90% of bladder tumors [1]. Clinicopathological characteristics of the bladder cancer such as tumor stage and grade have limitations in detecting and predicting high-risk disease progression [2]. Till date, the standard diagnostic methods for the identification and monitoring for recurrence and progression of bladder cancer are cystoscopy [3] and urine cytology [4]. The application of these gold standard diagnostic methods is limited in the routine clinical practice due to invasive nature and cost of cystoscopy and low sensitivity of urine cytology. Moreover, lack of infrastructure and medical facilities increases the mortality and morbidity in economically developing countries. Therefore, there is necessity for the development of biomarkers and therapeutic targets for better management of bladder TCC.

Multiple categories of tumor markers have been identified in the bladder cancer; but none of these markers are currently being in clinical use and their validation remains to be confirmed. Over the decades, various categories of tumor antigens were found but recently, a new category of tumor antigens, Cancer-testis (CT) antigens, are considered as potential antigen targets for early detection and cancer immunotherapy [5]. Recently, we identified and characterized a new member of CT antigen family; sperm associated antigen 9 (SPAG9) and demonstrated its involvement in mitogen-activated protein kinase (MAPK) signaling pathway [6]. SPAG9 functions as a scaffolding protein involved in c-Jun NH_2_-terminal kinase (JNK)-signaling module [6]. The JNK has been proposed to play an important role in cell survival, proliferation and tumorigenesis [7]. SPAG9 expression was shown to be associated with epithelial ovarian cancer [8], renal cell carcinoma [9], breast cancer [10], cervix cancer [11], thyroid cancer [12] and colorectal carcinoma [13]. These observations and findings are suggestive of SPAG9 as potential target for the development of diagnostic and therapeutic interventions. In light of these findings, SPAG9 expression and humoral response was investigated in bladder TCC patients. In addition, effect of *SPAG9* gene silencing on cellular proliferation, cell cycle, invasion and migration was assessed in highly invasive bladder cancer cell line, UM-UC-3.

## Materials and Methods

### Patient’s Specimens and Ethics Statement

A total of 125 bladder cancer patients tissues (male: 105; female: 20; median age 45 years, range 25–64 years) and 45 matched adjacent non-cancerous tissue (ANCT) specimens were obtained from Department of Urology, All India Institute of Medical Sciences, New Delhi, India, in accordance with local Ethics Committee. The approval for conducting the research was obtained from the Institutional Human Ethical Committee of All India Institute of Medical Sciences, New Delhi and National Institute of Immunology, New Delhi, India. Tissues were obtained after patient’s written consent; 67 patient’s specimens who underwent TURBT for the treatment of Ta, Tis and/or T1; and 58 patients who underwent radical cystectomy for the treatment of T2, T3. Immediately after surgical removal, all tissue samples were initially stored in “RNAlater” (Ambion, Austin, USA) and subsequently snap-frozen at −70°C. Pathologic reports were provided by the organisation for tissue. For accurate histopathological diagnosis, additional tumor specimens were formalin-fixed and paraffin-embedded. The 2004 WHO bladder tumor classification criteria (low grade and high grade) were used for grading [14] and pathologic staging was done according to the 2002 tumor-lymph node-metastasis classification system [15]. Detailed clinical characteristics of the patients are listed in Table 1. In addition, tissue sections were stained with hematoxylin and eosin and examined by 2 independent pathologists to confirm the diagnosis, typing and grading of tumor. None of the cases have received any therapy prior to surgery. Human sera were also obtained from 125 bladder TCC patients and from 50 normal healthy donors.

**Table 1:** Demographic and clinicopathological characteristics of bladder TCC patients.

	Total	RT-PCR/IHC (%)	ELISA (%)
**Total**	125	101/125 (81)	96/125 (77)
**ANCT**	45	0/45 (0)	
**Gender**			
Male	105		
Female	20		
**Pathologic tumor stage**			
Stage Ta	18	12/18 (67)	12/18 (67)
Stage Tis	06	06/06 (100)	06/06 (100)
Stage T1	44	38/44 (86)	35/44 (80)
Stage T2	25	17/25 (68)	15/25 (60)
Stage T3	32	28/32 (88)	28/32 (88)
**Superficial non-muscle invasive tumors**			
(Ta+Tis+T1)	68	56/68 (82)	53/68 (78)
**Muscle-invasive tumors**			
(T2+T3)	57	45/57 (79)	43/57 (75)
**Pathologic tumor grade**			
Low grade	50	40/50 (80)	40/50 (80)
High grade	75	61/75 (81)	56/75 (75)

### Cell Lines

Four human bladder cancer cells of different histological types, well differentiated HTB-2, moderately differentiated HTB-9, poorly differentiated HTB-1 and high grade invasive UM-UC-3 were procured from American Type Culture Collection (ATCC), and were used for all *in vitro* studies. All the cancer cell lines were cultured in minimal essential medium (MEM) supplemented with 10% heat-inactivated fetal calf serum (FCS) and 50 mg/ml Gentamycin, 100 mg/ml Streptomycin (Invitrogen, Carlsbad, CA) under standard conditions. As a control, Normal human urothelial (NHU) cell line was used and maintained as described earlier [16]. Although cell lines received from ATCC were not authenticated by the investigators, however cells were grown immediately and stored in liquid N_2_. Cells were cultured and used for various experiments and were not cultured more than 5 months after the cell lines were revived. All cell lines were regularly monitored and were found to be mycoplasma free.

### RT-PCR and Western Blot Analysis

Total RNA was isolated from 50–100 mg of tissue specimen or 1×10^6^ cancer cells using TRI reagent solution (Ambion Inc., Austin, TX) and RT-PCR was carried out as described earlier [10]. The sequences of *SPAG9* primers used in the present investigation were: *SPAG9* forward 5′-GACAGAGATGATTCGGGCATCACGAGAAAA-3′ and *SPAG9* reverse 5′-CTAAGTTGATGACCCATTATTATACCTCGACTG-3′. The amplified product was cloned in TOPO TA cloning vector (Invitrogen, Carlsbad, CA) and sequenced. *β-Actin* primers were used as positive control. SPAG9 protein expression in different cell lines was analysed by probing 40 µg whole cell lysate with anti-SPAG9 antibody as described earlier [11].

### *In situ* RNA Hybridization

*In situ* RNA hybridization was carried out in bladder TCC tissue specimens by employing *in vitro* synthesized RNA probes (riboprobes) as described earlier [11]. All tissue specimens were processed for *in situ* RNA hybridization under RNase free conditions. Briefly, sections were deparaffinized, rehydrated and were probed using sense riboprobes (control) and antisense riboprobe (experimental) following the protocols supplied with Digoxigenin RNA Labeling and detection kit (Roche applied Sciences, Indianapolis).

### Immunohistochemistry

Immunohistochemistry (IHC) experiments were performed in serial sections of bladder TCC tissue samples and ANCT using anti-SPAG9 antibody raised in rat against recombinant SPAG9 protein as described earlier [13]. In addition, to demonstrate the specificity of anti-SPAG9 antibody raised in rats, anti-SPAG9 antibody was neutralized by pre-incubating anti-SPAG9 antibody with 15 µg/ml of recombinant SPAG9 protein and subsequently used for IHC. SPAG9 immunoreactivity score (IRS) was assessed by counting 500 cells from 5 random fields of each specimen under ×400 magnification in SPAG9 reactive tumor area of each section by senior pathologist in superficial non-muscle invasive (pTa, Tis and T1) and muscle invasive (T2 and T3) bladder tissue sections, as described earlier [16]. We considered a distinct positive immunoreactivity in a specimen showing >10% of cancer cells stained for SPAG9 protein. Further, TCC specimens were classified as SPAG9 IRS group I showing >60% SPAG9 positive cells and SPAG9 IRS group II showing <60% SPAG9 positive cells.

### ELISA and Immunoblotting

The humoral response in serum of bladder cancer patients and in normal healthy donors was detected by ELISA and Western blotting using recombinant SPAG9 protein, as described previously [10]. The cut-off value was calculated by mean+2 SD of antibody titers of healthy donors. For validating the humoral response, 0.5 µg SPAG9 protein was probed with patient’s sera (1∶500), followed by incubation with anti-human IgG antibody (Jackson ImmunoResearch, West Grove, PA, USA). The color was developed by using 3, 3′-diaminobenzidine as a substrate. Specificity of the anti-SPAG9 antibodies in patient’s sera was confirmed by neutralizing the patient’s sera with 15 µg/ml of recombinant SPAG9 protein. The neutralized patient’s sera were subsequently used for Western blot analysis.

### Immunofluorescence Microscopy and Flow-cytometric Analysis

For immunofluorescence assay, HTB-2, HTB-9, HTB-1 and UM-UC-3 cells were seeded at a density of 1×10^5^ cells/coverslip and grown in a six-well tissue culture plate for 16 h. After fixing cells with 3% (w/v) paraformaldehyde in PBS, cells were permeabilized with 0.5% IGEPAL (Sigma), and blocked with 5% normal goat sera and were processed for immunofluorescence assay as described previously [9]. For examining distribution of SPAG9 protein in different cellular compartments, cells were probed with antibodies of mitochondria (MTCO2, mitochondrial marker, ab3298; Abcam), endoplasmic reticulum (calnexin, 6D195, ER marker, sc-70481; Santa Cruz Biotechnology, Santa Cruz, CA), golgi body (GM130 B-10, Golgi body marker, sc-55591; Santa Cruz Biotechnology), and nuclear envelop (lamin A/C 636, nuclear envelop marker, sc-7292; Santa Cruz Biotechnology) respectively as described earlier [13]. The fluorescence images were merged using Image Pro-Plus, version 6.1.

For flow cytometric analysis, HTB-1, HTB-2, HTB-9 and UM-UC-3 cells (1×10^6^) were harvested and fixed with 3% (w/v) paraformaldehyde in PBS, washed twice with PBS and incubated with rat anti-SPAG9 antibodies [9]. Cells were washed with PBS (thrice) and incubated with goat anti-rat IgG-FITC conjugate (Jackson ImmunoResearch, West Grove, PA, USA). After the final wash, SPAG9 expression was observed by running the samples on an BD LSR cytometer (BD Biosciences) and data analysed using WinMDI (version 2.8) software [9].

### RNA Interference and Transient Transfection

In order to perform gene silencing experiments, two independent sets of siRNA target sequences directed against *SPAG9* cloned BS/U6 vector were used (designated SPAG9 siRNA-I and SPAG9 siRNA), alongwith control siRNA (scrambled SPAG9), as described previously [9]. The efficiency of the constructs was tested through transfection into UM-UC-3 cell line and Western blot analysis of the total cell lysates with the SPAG9 antibodies. SPAG9 siRNA-I, SPAG9 siRNA or scrambled siRNA transfection was performed in opti-MEM with the transfection reagent Lipofectamine^Tm^ Plus (Invitrogen Life Technologies, Carlsbad, CA) as previously described [13].

### Cell Cycle Analysis

SPAG9 siRNA or scrambled siRNA transfected UM-UC-3 cells were harvested and fixed in chilled 70% ethanol for 1 h, stained with propidium iodide (PI; 18 µg/ml) containing 400 µg/ml RNaseA with shaking for 1 h, and analyzed by flow cytometry for cell cycle profile. The SPAG9 siRNA induced G_0_–G_1_ arrest was characterized by probing the SPAG9 siRNA transfected UM-UC-3 cell lysate with antibodies against cyclin B, cyclin D, cyclin E, p16, p21, CDK1 and CDK4. Antibodies against β-actin, cyclin B, cyclin D, cyclin E, p16 and p21 were purchased from Santa Cruz Biotechnology, Inc. *(*Santa Cruz, CA). Antibodies against CDK1 and CDK4 were procured from Abcam (Cambridge, MA). All experiments were done at least thrice in duplicates.

### Cellular Proliferation Analysis

The effects of down-regulation of SPAG9 expression using SPAG9 siRNA on proliferation in UM-UC-3 cells were investigated by using chromogenic dye 3-(4,5-dimethylthiazol-2-yl)-2, 5-diphenyltetrazolium bromide (MTT, sigma-aldrich). Absorbance at 570 nm-650 nm was recorded on ELISA plate reader (Molecular Devices, Sunnyvale, CA).

### Cell Invasion, Migration, and Wound Healing Assay

The effects of SPAG9 siRNA on cell migration, invasion, and wound healing were assessed as described previously [13]. Briefly, for migration assay, 2×10^4^ SPAG9 siRNA or control siRNA transfected cells were seeded on to 8-µm pore inserts in 24-well plate. After 24 hours, cells migrated to the lower chamber were fixed with 5% glutaraldehyde and subsequently stained with 0.5% toluidine blue, and cell number was calculated by microscopy. Similarly, for invasion assay, inserts were coated with 15 µg matrigel (Becton Dickinson Labware, Bedford) and the same protocol was followed as that of migration assay. For wound healing assay, 2×10^6^ cells were seeded in 35 mm petri dish. Post 12 h of incubation, a wound was mechanically created using an aerosol P200 pipette tip, and photomicrographed at various time points to examine the effect of SPAG9 down-regulation on cell migration.

### Statistical Analysis

Mann Whitney test and Pearson’s Chi-Square test were performed using SPSS 20.0 statistical software package (SPSS Inc, Chicago, IL, USA). Data are expressed as mean±standard error of at least three independent experiments in *in vitro* assays. All *P* values were 2-sided and a *P*-value of less than 0.05 was considered statistically significant.

## Results

### *SPAG9* Gene Expression in Bladder TCC Tissue Specimens

**Figure 1:**
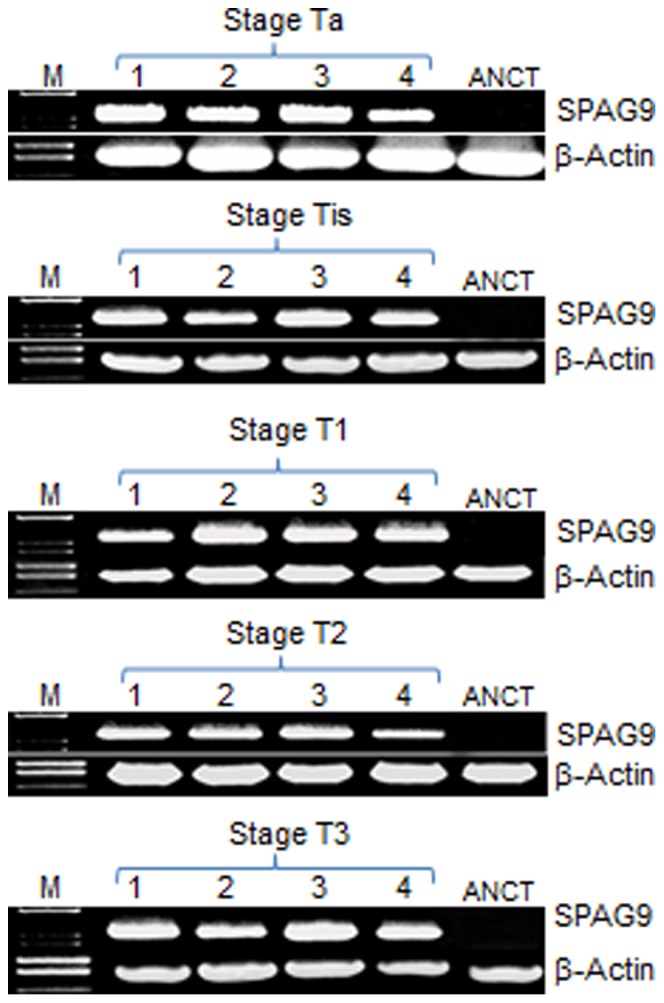
*SPAG9* gene expression in bladder TCC patient’s tissue specimens. RT-PCR analysis of *SPAG9* mRNA showing specific SPAG9 PCR amplicon in representative patient’s specimen of stage Ta, Tis, T1, T2 and T3 (Lane 1–4). No *SPAG9* expression was detected in matched ANCT specimens (Lane 5). Lane M - molecular size marker. *β-Actin* gene expression was used as an internal control.

*SPAG9* mRNA was detected in 81% bladder TCC tissue specimens by employing RT-PCR among which 82% were of superficial non-muscle invasive and 79% were of muscle-invasive tumor specimens (Table 1). In addition, *SPAG9* expression was detected in 80% patients with low grade and 81% patients with high grade bladder tumors (Table 1). For each tumor stage of bladder TCC, representative RT-PCR results are shown in Figure 1. *SPAG9* mRNA expression was not observed in matched ANCT tissues. PCR product was sub-cloned and sequenced which showed no mutation in the *SPAG9* gene.

**Figure 2:**
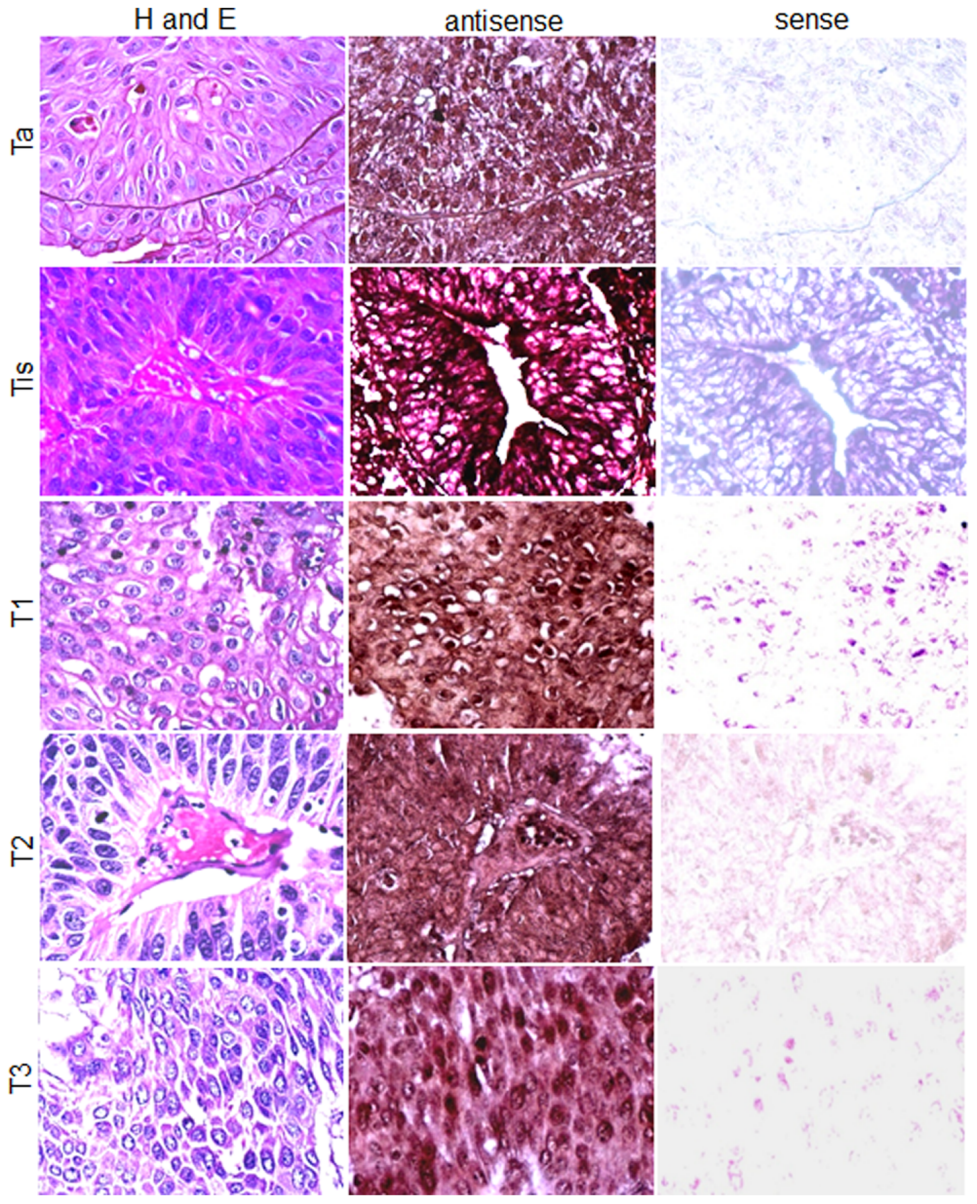
Cell type *SPAG9* gene expression in bladder TCC patient’s tissues. Representative H&E stained tissue specimens showing cytostructure of Ta, Tis, T1, T2 and T3 stages (left panel). *In situ* RNA hybridization showing SPAG9 expression in matched serial tissue section from same specimens of Ta, Tis, T1, T2 and T3 stages showed a strong chocolate brown color with digoxigenin-labeled *SPAG9* antisense riboprobes (middle panel). However, *SPAG9* sense riboprobe failed to show the signal (right panel). Original magnification, ×400; objective, 40×.

Subsequently, cell specific *SPAG9* gene expression was determined by employing *in situ* RNA hybridization in serial sections of different tumor stages. Antisense *SPAG9* riboprobe resulted in chocolate brown reaction product, indicating the presence of *SPAG9* mRNA in cells of bladder TCC tissues (Figure 2). However, the *SPAG9* sense riboprobe failed to show any localization. These results indicated that 81% of total bladder TCC specimens revealed *SPAG9* gene expression (Table 1).

### SPAG9 Protein Expression in Bladder TCC using IHC

**Figure 3:**
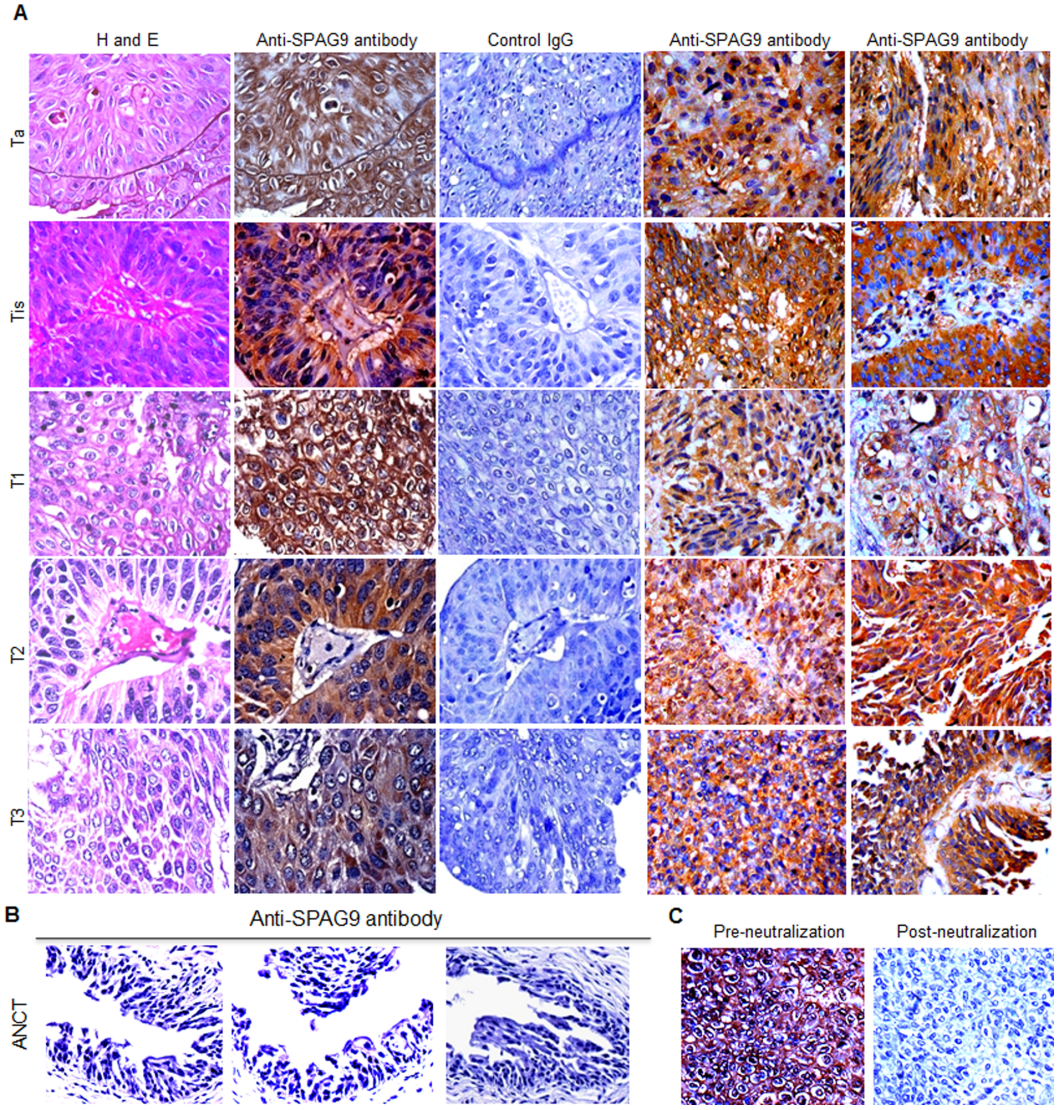
Validation of SPAG9 protein expression in bladder TCC patient’s tissue. (**A**) Representative H&E stained tissue specimens of Ta, Tis, T1, T2 and T3 stages (I panel). Immunohistochemical analysis of SPAG9 protein expression in matched serial tissue section from the same specimens of Ta, Tis, T1, T2 and T3 stages probed with anti-SPAG9 antibodies showed SPAG9 cytoplasmic localization (II panel). Control IgG showed no reactivity (III panel). Panel IV and V representing different tissue specimens sections of Ta, Tis, T1, T2 and T3 stages probed with anti-SPAG9 antibodies showed SPAG9 protein expression (**B**) Matched ANCT specimens probed with anti-SPAG9 antibodies showed no reactivity. (**C**) Representative photomicrographs showing immunoreactivity of anti-SPAG9 antibody before and after neutralization. Neutralization of anti-SPAG9 antibody raised in rats revealed loss of immunoreactivity in IHC which confirms the specificity of anti-SPAG9 antibody. Original magnification, ×400; objective, 40×.

SPAG9 protein validation was carried out on serial sections of tumor specimens by IHC. SPAG9 expression was detected in 81% bladder TCC patients. Interestingly, 82% of superficial non-muscle invasive and 79% of muscle-invasive tumor specimens revealed SPAG9 expression (Table 1). In addition, SPAG9 expression was detected in 80% patients with low grade and 81% patients with high grade bladder tumors (Table 1). Representative photomicrographs of bladder TCC serial sections showed cytoplasmic localization of SPAG9 protein in tumor cells of various stages (Figure 3A). However, matched serial section of tumor tissue probed with control IgG showed no reactivity (Figure 3A). As shown in representative photomicrograph, no reactivity was observed in ANCT specimens probed with anti-SPAG9 antibody (Figure 3B). Further, to confirm the specificity of anti-SPAG9 antibody raised in rats, anti-SPAG9 antibody was neutralized by pre-incubating with 15 µg/ml of recombinant purified SPAG9 protein. The neutralized anti-SPAG9 antibody was subsequently used for IHC in clinical specimens. The IHC results revealed complete loss of immunoreactivity as shown in representative bladder TCC specimens (Figure 3C).

**Figure 4:**
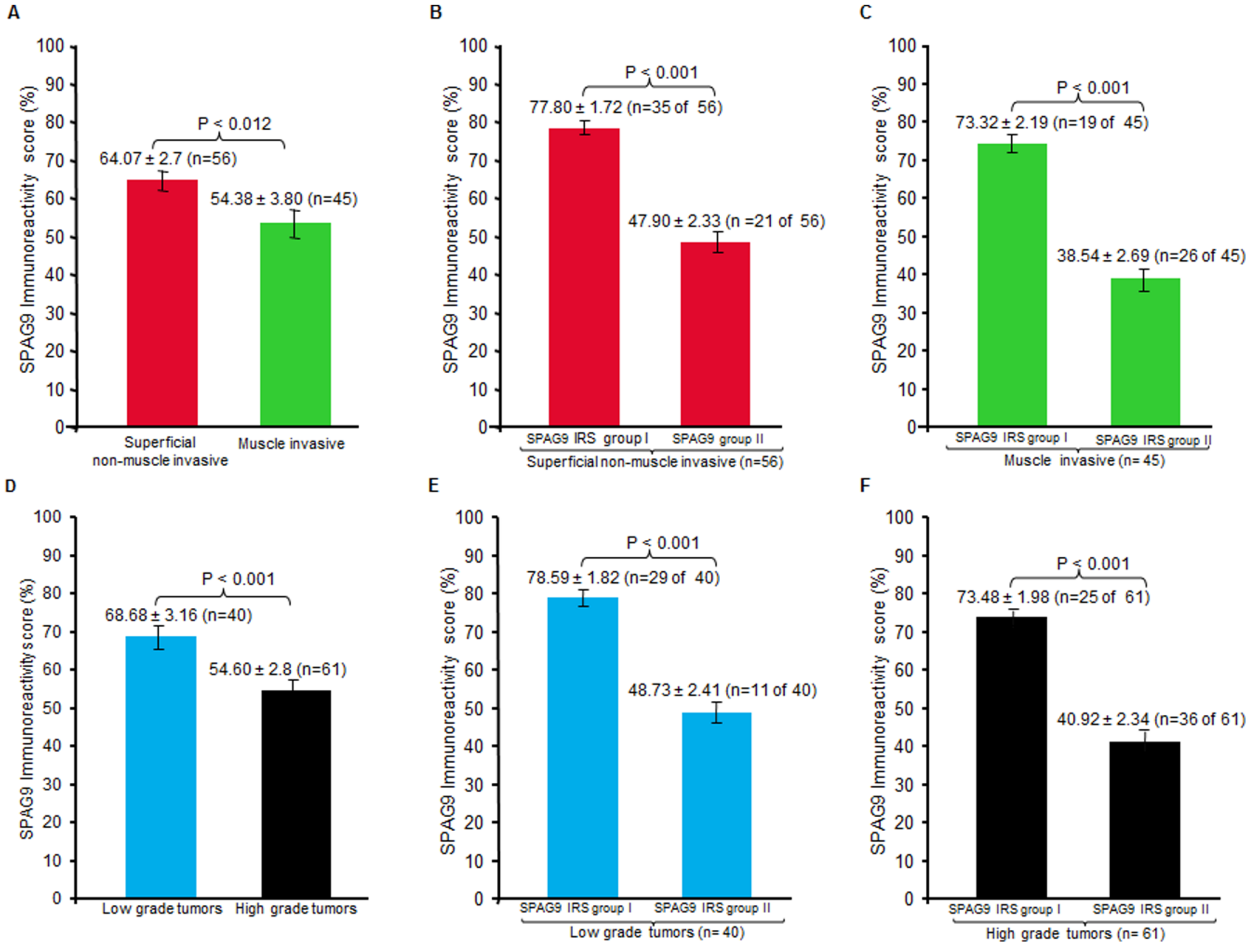
SPAG9 Immunoreactivity score (IRS) in different tumor stages and grades of bladder TCC patients. (**A**) SPAG9 IRS in superficial non-muscle invasive tumors and in muscle-invasive tumors revealed significant difference (*P*<0.012). (**B**) As depicted in histograph, analysis of SPAG9 expression, amongst superficial non-muscle invasive tumor patients was found to be significantly different (P<0.001) in SPAG9 IRS group I (>60% SPAG9 positive cells; n = 35 of 56) and SPAG9 IRS group II (<60% SPAG9 positive cells; n = 21 of 56). (**C**) Muscle invasive tumor patients, a significant difference (P<0.001) of SPAG9 IRS was observed in the two groups: SPAG9 IRS group I (>60% SPAG9 positive cells; n = 19 of 45) and SPAG9 IRS group II (<60% SPAG9 positive cells; n = 26 of 45) as shown in histograph. (**D**) There was significant difference in SPAG9 IRS in low and high grade tumor tissue specimens (*P*<0.001). (**E**) As shown in histograph, analysis of SPAG9 expression within low grade tumors grade significant difference (P<0.001) was found between SPAG9 IRS group I (>60% SPAG9 positive cells; n = 29 of 40) and SPAG9 IRS group II (<60% SPAG9 positive cells; n = 11 of 40). (**F**) As shown in histograph, SPAG9 expression was found to be significantly different (P<0.001) within high grade tumor patients between SPAG9 IRS group I (>60% SPAG9 positive cells; n = 25 of 61) and SPAG9 IRS group II (<60% SPAG9 positive cells; n = 36 of 61). Point indicates mean; bar, standard error.

Although 81% of patients showed SPAG9 protein expression, SPAG9 expression was heterogeneous in terms of number of cells expressing SPAG9 protein. Interestingly, SPAG9 expression was found to be associated with cancerous tissues (P<0.001), using Pearson chi- square test, as none of the ANCT specimens revealed detectable SPAG9 immunoreactivity. No significant association of SPAG9 expression with tumor stages (P = 0.311) and grades (P = 1.00) was found by Pearson’s Chi-Square test. Based on SPAG9 IRS, there was significant difference of SPAG9 expression found between the superficial non-muscle invasive and muscle-invasive tumors by using Mann Whitney test (P = 0.012; Figure 4A).

We further analysed SPAG9 IRS group I (>60% SPAG9 positive cells) and SPAG9 IRS group II (<60% SPAG9 positive cells) within different stages. Interestingly, a significant difference was found between two groups in superficial non-muscle invasive (P<0.001; Figure 4B) with a higher number of patients (n = 35 of 56) belonging to SPAG9 IRS group I (>60% SPAG9 positive cells), as assessed by Mann Whitney test. Similarly, there was a significant difference found in SPAG9 IRS muscle-invasive tumors group I (>60% SPAG9 positive cells; n = 19 of 45) and group II (<60% SPAG9 positive cells; n = 26 of 45) employing Mann Whitney test (P<0.001; Figure 4C). Collectively, high SPAG9 IRS (>60% SPAG9 expressing cells) was found to be significantly associated with superficial non-muscle invasive tumors using Pearson’s chi-square test (P = 0.042) indicating the role of SPAG9 in early tumorigenesis.

Among grades, there was significant difference found among low and high grade tumors by employing Mann-Whitney test (P<0.001; Figure 4D). When SPAG9 IRS group I (>60% SPAG9 positive cells) and SPAG9 IRS group II (<60% SPAG9 positive cells) were compared within low grades, a significant difference was observed (P<0.001), with a higher number of specimens (n = 29 of 40) demonstrating >60% SPAG9 positive cells, as examined by Mann-Whitney test (Figure 4E). Similarly, when SPAG9 IRS group I (n = 25 of 61) and SPAG9 IRS group II (n = 36 of 61) were compared within high grades, a significant difference was observed (P<0.001) employing Mann-Whitney test (Figure 4F). In summary, high SPAG9 IRS (>60% SPAG9 positive cells) was found to be significantly associated with low grades, as compared to high grades, by using Pearson’s chi-square test (P = 0.002). Overall, association of high SPAG9 IRS (>60% SPAG9 positive cells) with early stages (superficial non-muscle invasive) and low grades suggests the potential role of SPAG9 in early tumorigenesis.

### Humoral Immune Response against SPAG9 Protein in Bladder TCC Patients

**Figure 5:**
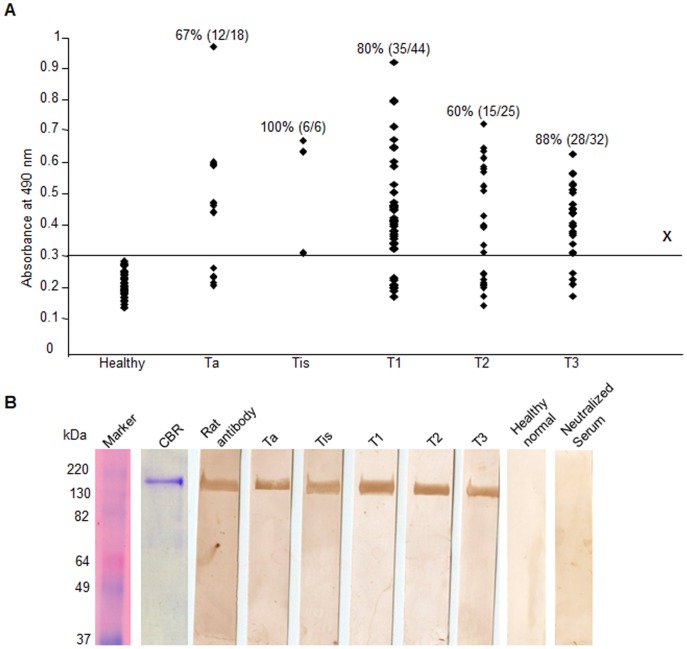
Humoral response against SPAG9 in bladder TCC patients. (**A**) Circulating anti-SPAG9 antibodies in sera from bladder TCC patients. Line X denotes cutoff value taken at 490 nm (mean+2 SD) from healthy donors (n = 50) for positivity above or negativity below the line. Marker- molecular size marker, CBR- Coomassie brilliant blue stained recombinant SPAG9 protein, Rat antibody- antibodies raised in rat against recombinant SPAG9 protein: positive control (**B**) Western blotting confirmed the circulating anti-SPAG9 antibodies in five representative patient’s serum. No reactivity detected in healthy donors. Neutralization experiments resulted in complete loss of immunoreactivity [patient’s sera preincubated with recombinant SPAG9 protein (15 µg/ml)].

Humoral response against SPAG9 was investigated in 125 bladder TCC patient’s sera. Fifty healthy blood donors matched for age with the cancer population were selected as a control group. Employing ELISA, the mean absorbance value at 490 nm of healthy donor, a cutoff value was determined by adding 2 SD [(0.234)+2 SD (0.035) = 0.304] (Figure 5A). All bladder TCC samples and normal healthy donor samples were tested in duplicates and the mean was used for data analysis. Positive immunoreactivity against SPAG9 protein was detected in 77% bladder TCC patients. Overall, 78% of patients with superficial non-muscle invasive and 75% of patients with muscle-invasive tumors showed reactivity against SPAG9 protein (Table 1). Similarly, 80% of low grade patients and 75% of high grade patients demonstrated SPAG9 antibodies (Table 1). No significant association of SPAG9 immune response was found among the tumor stages (*P* = 0.486) and tumor grades (*P* = 0.669) by Pearson’s Chi-Square test. Our results indicated that humoral immune response against SPAG9 was independent of various tumor stages and grades.

To further confirm the circulating anti-SPAG9 antibodies in the bladder TCC patients’ sera, Western blot was done using recombinant SPAG9 protein (Figure 5B) which revealed that immunoreactivity was found in 77% of total bladder TCC patients irrespective of tumor stages (Table 1). Representative blot from each tumor stage are shown in Figure 5B. In the neutralization experiment, patient’s serum was pre-incubated with 15 µg/ml of recombinant SPAG9 protein which resulted in complete loss of reactivity.

### SPAG9 Expression in Bladder Cancer Cell Lines

**Figure 6:**
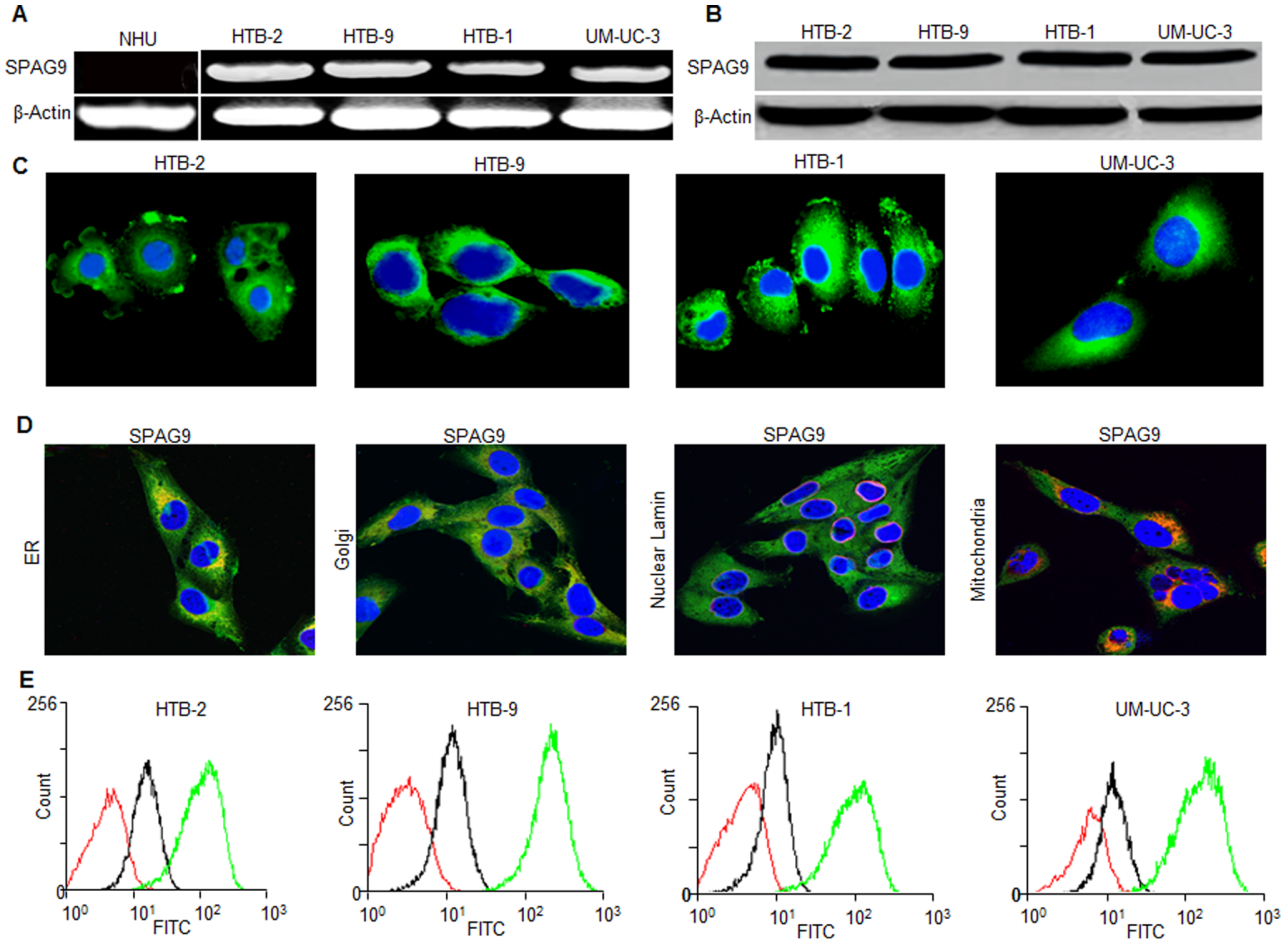
SPAG9 expression in bladder cancer cell lines. (**A**) *SPAG9* mRNA expression assessed by RT-PCR revealed the presence of *SPAG9* transcript in all bladder cancer cells. However, NHU cells did not reveal *SPAG9* transcript. *β-Actin* was used as an internal control. (**B**) Western blot analysis of SPAG9 protein expression. (**C**) Indirect immunofluorescence revealed SPAG9 cytoplasmic localization in all bladder cancer cells. Nuclear staining was done by DAPI. (**D**) UM-UC-3 cancer cells were probed for SPAG9 and various markers for cell organelles. Co-localization of SPAG9 was examined by indirect immunofluorescence assay which revealed SPAG9 co-localization with ER and Golgi marker. Nuclear envelop and mitochondria did not show co-localization with SPAG9. Immunofluorescence staining was detected by a laser-scanning confocal microscope. The images are merged for co-localization of SPAG9 (green) and marker co-staining (red). Original magnification, ×630; objective, 63×). (**E**) Flow cytometric analysis demonstrated surface localization of SPAG9 protein in fixed cancer cells (green histogram) compared with cells probed with control IgG (black histogram) or stained with secondary antibody only (red histogram). Results from 1 of 3 representative experiments are shown.

SPAG9 gene and protein expression was investigated in HTB-2, HTB-9, HTB-1 and UM-UC-3 bladder TCC cells. RT-PCR analysis revealed that the *SPAG9* mRNA expression was detected in all cancer cell lines but not in NHU cell line (Figure 6A). The PCR product in cancer cells showed no mutations by DNA sequence analysis. Further, we investigated SPAG9 protein expression employing Western blotting which confirmed SPAG9 expression in all cancer cells (Figure 6B). Our indirect immunofluorescence microscopy results showed that SPAG9 protein was localized in the cytoplasm of HTB-2, HTB-9, HTB-1 and UM-UC-3 (Figure 6C). We further probed SPAG9 co-localization with various cell organelles by using multiple markers for Golgi body (GM130), endoplasmic reticulum (calnexin), nuclear envelope (lamin A/C) and mitochondria (MTCO2) in high grade invasive UM-UC-3 cancer cells. Our data clearly showed SPAG9 co-localization with Golgi marker and ER marker (Figure 6D). However, SPAG9 did not appear to co-localize directly with mitochondria marker or with nuclear lamin (Figure 6D). Subsequently, in fixed cancer cells, flow cytometric analysis revealed significant increase in displacement of fluorescence on the x axis when probed with anti-SPAG9 antibody indicating surface localization of SPAG9 protein in all cancer cells (Figure 6E; green histogram). However, cells probed with control IgG showed no or very low surface distribution or fluorescence intensity (Figure 6D, black histogram). These results indicated that SPAG9 has distinct cytoplasmic and surface localization and co-localization with Golgi and ER.

### Silencing of *SPAG9* Gene in Human Bladder Cancer Cells

**Figure 7:**
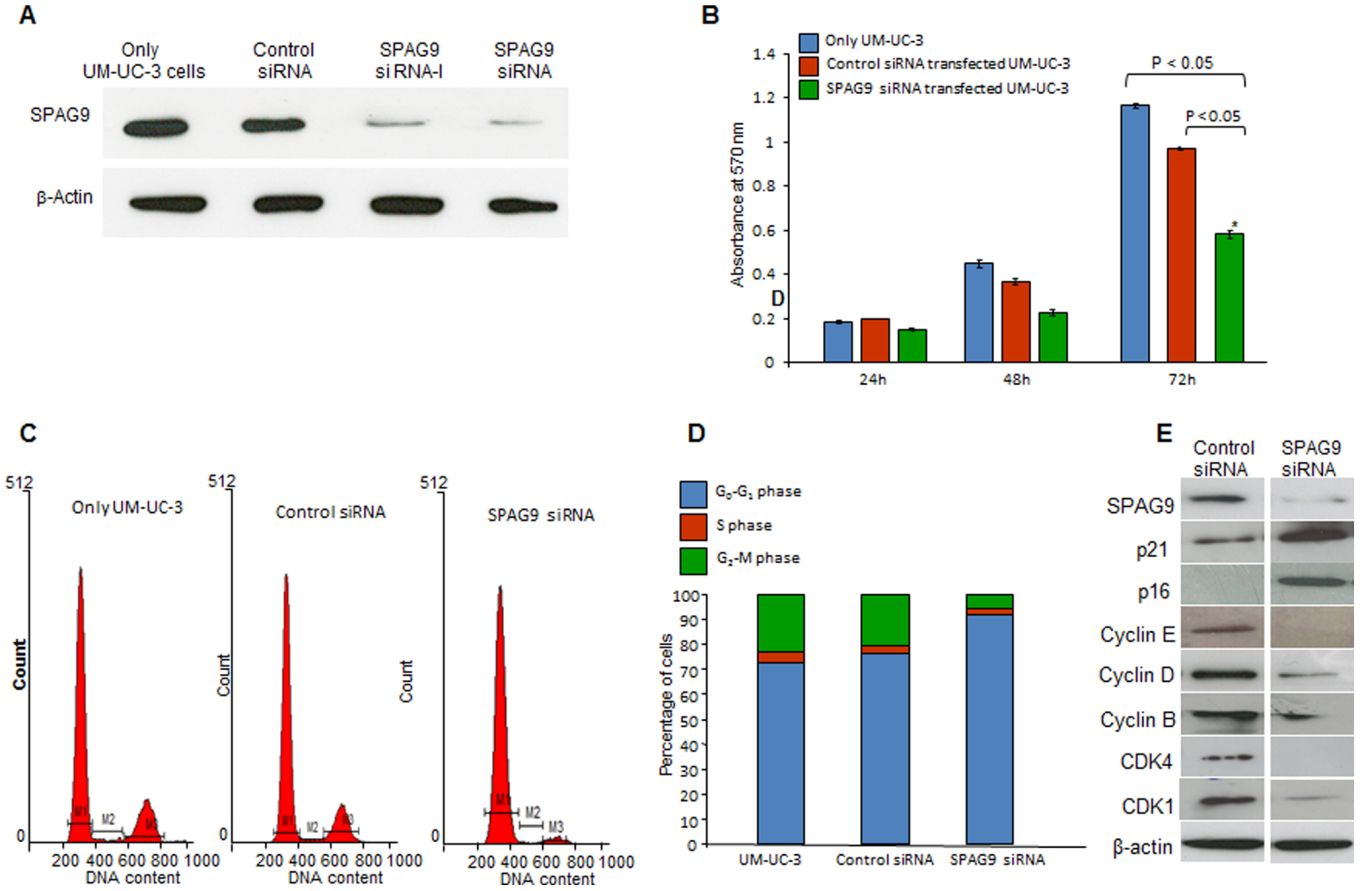
*SPAG9* Silencing and its effect on cellular proliferation and cell cycle. (**A**) Western blot depicts ablation of SPAG9 protein in UM-UC-3 cells transfected with SPAG9 siRNA. β-Actin was used as a control. (**B**) Knockdown of *SPAG9* by siRNA inhibited cell growth in UM-UC-3 cells *in vitro* determined by MTT assay. (**C**) Silencing of *SPAG9* expression arrested cell cycle progression in G_0_–G_1_ phase in SPAG9 siRNA transfected cells as compared with only cells or scrambled siRNA transfected cells. (**D**) Histogram analysis confirmed that in SPAG9 siRNA transfected cells accumulated in G_0_–G_1_ phase. (**E**) Western blot analysis of cell cycle molecules in SPAG9 ablated UM-UC-3 cells depicted up-regulation of CDK inhibitors including p21 and p16. The up-regulation of p21 and p16 was preceded by down-regulation of cyclin E, cyclin D, cyclin B, CDK4 and CDK1. β-Actin was used as an internal loading control. [M1- G_0_–G_1_ phase_;_ M2- S phase and M3- G_2_-M phase].

Gene silencing approach was employed to knockdown the *SPAG9* expression in high-grade invasive bladder cancer cell UM-UC-3. The vector-based siRNA plasmids (SPAG9 siRNA- I, SPAG9 siRNA, and control siRNA), were used for transient transfections in the UM-UC-3 cells. After 72 h of transfections, Western blot analysis demonstrated the ablation of SPAG9 protein expression in UM-UC-3 cells transfected with SPAG9 siRNA-I and SPAG9 siRNA as compared to only UM-UC-3 or UM-UC-3 cells transfected with control siRNA (Figure 7A). Therefore, for all subsequent experiments were restricted to SPAG9 siRNA for rest of the gene silencing studies. In order to investigate the potential role of SPAG9 protein in cellular proliferation, MTT assay was carried out at 24 h, 48 h, and 72 h post-transfection. MTT assays revealed that there was significant suppression in the growth of UM-UC-3 cells when transfected with SPAG9 siRNA (*P*<0.05; Figure 7B). In contrast, no suppressive effect on the growth of UM-UC-3 cells was observed in scrambled siRNA transfected cells (Figure 7B). Also as shown in the histogram analysis 72 h post transfection, the inhibitory effect by SPAG9 siRNA was found to be significantly higher as compared to scrambled siRNA transfected cells (*P*<0.05; Figure 7B). These results suggest that SPAG9 may have role in cellular proliferation.

### Cell Cycle Arrest of UM-UC-3 Cells by Suppression of *SPAG9* Expression

To explore the underlying mechanism by which *SPAG9* expression promotes bladder cancer cellular proliferation, cell cycle analysis was carried out to investigate whether SPAG9 siRNA transfection affected the cell cycle in UM-UC-3 cells. As shown in Figure 7C, the percentage of cells in the G_2_-M phase decreased markedly and the percentage of cells in the G_0_–G_1_ phase increased markedly in the population of cells with ablated SPAG9 protein expression compared with only cells or cells transfected with scrambled siRNA. The histogram analysis demonstrated that the percentage of cells at G_0_–G_1_ phase increased by 25.67% in the UM-UC-3 cells transfected with the SPAG9 siRNA plasmid while the percentage of cells at M phase decreased by 15.66% (Figure 7D). We further validated the G_0_–G_1_ growth arrest at the molecular level by carrying out Western blot analysis for proteins involved in induction of G_0_–G_1_ arrest in SPAG9 ablated UM-UC-3 cells. Importantly, our data showed that SPAG9 siRNA induced G_0_–G_1_ arrest was accompanied by significant reduction in CDK inhibitors including p21 and p16. Further, the increased levels of CDK inhibitors such as p21 and p16 were correlated with down-regulation of cyclin E, cyclin D, cyclin B, CDK1 and CDK4 (Figure 7E). Taken together, these results suggest that ablation of SPAG9 protein inhibited cellular proliferation by inducing G_0_–G_1_ growth arrest in high grade invasive urothelial UM-UC-3 cells.

### Effect of *SPAG9* Knockdown on Migration and Invasion of Bladder Cancer Cell

**Figure 8:**
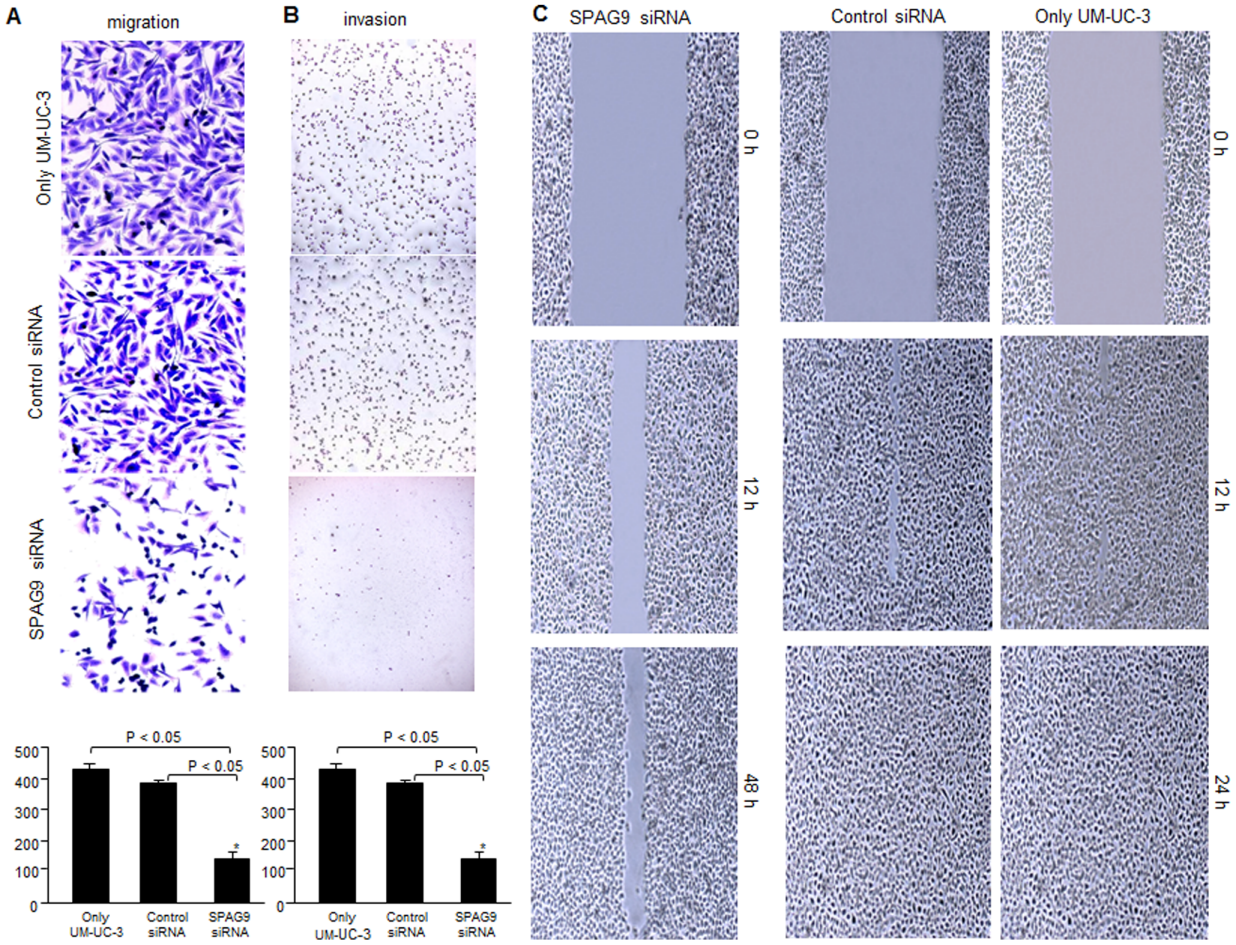
Effect of SPAG9 expression on migration and invasion ability of UM-UC-3 cells. Knockdown of SPAG9 expression inhibited (**A**) Migration, (**B**) Invasion and (**C**) Wound healing assay are depicted in only cells scrambled siRNA and SPAG9 siRNA transfected cells.

The migration assay analysis demonstrated that SPAG9 depletion caused a significant decrease in the number of migrating cells compared to only UM-UC-3 or scrambled siRNA transfected UM-UC-3 cells (Figure 8A). The histogram analysis of migration assay revealed a significant reduction in number of cells migrated through inserts (*P*<0.05) in SPAG9 siRNA transfected UM-UC-3 cells as compared to only UM-UC-3 or scrambled siRNA transfected UM-UC-3 cells (Figure 8A). Similar results were obtained in invasion assay in which cells cross a reconstituted basement membrane of matrigel coated on top of transwell filters (Figure 8B). Further, histogram representation of matrigel invasion assay demonstrated that the number of invading cells in the lower chamber was significantly reduced in *SPAG9* knockdown UM-UC-3 cells compared to only UM-UC-3 cells or scrambled siRNA transfected cells (*P*<0.05; Figure 8B).

The effect of *SPAG9* knockdown on the migration potential of UM-UC-3 cells was further evaluated in wound healing assay. Confluent monolayers of SPAG9 siRNA and scrambled siRNA transfected UM-UC-3 or only UM-UC-3 cells were disrupted (i.e., mechanically wound was created by scraping with a 200 µl pipette tip).Wound closure was followed at 12 h, 24 h and 48 h. Within 24 h, the area of the wound was significantly recovered in only UM-UC-3 cells or in UM-UC-3 cells transfected with scrambled siRNA (Figure 8C). In contrast, wound closure of SPAG9 siRNA transfected UM-UC-3 cells was not completed even after 48 h (Figure 8C). These data indicate that *SPAG9* gene expression plays an important role in bladder cancer cell migration.

## Discussion

Bladder cancer contributes significantly to the overall human cancer burden being the second most common malignancy of genitourinary tract with a high recurrence rate and poor prognosis [1]. The frequent recurrence of superficial non-muscle invasive tumors and distant metastasis of muscle-invasive tumors are major problems in clinical management of bladder cancer. Thus, there is a need to identify new tumor specific biomarkers for early diagnosis and prediction of bladder TCC for an appropriate cancer management of the patients.

Cancer-testis (CT) antigens are protein antigens with normal expression restricted to testicular germ cells, and yet are aberrantly expressed in various cancerous tissues [17]. With the tumor-associated expression pattern, CT antigens have become a prime focus for early detection strategies against cancer in recent years. Although a number of CT antigens have been discovered and has been suggested to be associated with the human malignancy [17], the complexity of carcinogenesis mechanism reflects on the need to establish compelling new criteria for validating their real applicability in cancer screening and therapeutics [18]. CT antigens in bladder TCC tissue specimens are among the least studied antigens. In the present study, SPAG9 expression in the human bladder TCC tissue specimens was investigated. *SPAG9* mRNA expression was detected in 81% of bladder TCC specimens. This predominant *SPAG9* gene expression in bladder TCC patients was found to be interesting when compared with other known potential CT antigens. Characterization of the expression of *MAGE-A* gene family in bladder cancer has demonstrated that members of the *MAGE-A* family are frequently expressed in bladder tumors at a level above the normal bladder tissue [19]–[21]. Yet, another well-characterized CT antigen *NY-ESO-1* mRNA expression was reported in only 45% of the bladder TCC specimens [20]. In contrast using RT-PCR and *in situ* RNA hybridization, *SPAG9* mRNA expression was observed in majority of bladder TCC tissues (81%) analyzed irrespective of tumor stages and grades. It was interesting to note that the patients with superficial non-muscle invasive tumors showed predominant *SPAG9* expression (82%) which suggests its role in bladder tumorigenesis. However, large studies are warranted to validate the findings.

The *SPAG9* gene expression was further validated for protein expression by IHC. Majority of bladder TCC patients (81%) showed SPAG9 protein expression by IHC. All the tumor tissue specimens found positive for *SPAG9* mRNA were also found positive for SPAG9 protein expression. Interestingly our data showed no discrepancy between RT-PCR analysis, *in situ* RNA hybridization and IHC. In contrast, protein expression of very few CT antigens has been reported in literature [19]–[21]. Bladder cancers of higher grade are likely to proceed towards the muscle invasion stage, which is directly related to mortality. In contrast, if tumor is confined to the lamina propria i.e., superficial non-muscle invasive tumors, can be cured in the majority of the patients with localized therapies [22]. In the present study, SPAG9 expression was observed in 82% of superficial non-muscle invasive bladder TCC specimens indicating that SPAG9 may be a target candidate molecule for early detection and diagnosis in bladder TCC.

We were intrigued by the observation that high SPAG9 IRS (>60% SPAG9 positive cells) were significantly associated with early stages (superficial non-muscle invasive) and low grade as compared to late stages (muscle-invasive) and high grades. This inverse correlation of SPAG9 expression and disease progression might suggest that SPAG9 is required in early spread of tumor progression, when cells acquire the malignant properties such as cellular proliferation, invasion and migration. Bladder cancer usually begins as a superficial protrusion that can be located at the mucosa (Ta tumors) or the submucosa (invasive lamina propria T1 tumors). More over carcinoma *in situ* lesions commonly represent the precursor stage of aggressive carcinoma. In this regard a recent report on PTPD1, a cytosolic non-receptor protein –tyrosine phosphatase showed abundant expression of PTPD1 in bladder lesions at early stages (pTa) than in the late advanced stage (T3) [23]. The inverse correlation between PTPD1 expression and disease progression was shown to reflect a requirement of PTPD1 in early step of tumor progression. Another study on Rb and p16 revealed similar distribution of expression detected by immunohistochemistry indicating higher expression in early stages (pTa/pT1) and grade 1 [24]. Our earlier study in bladder urothelial cancer revealed significant association of higher HSP70-2 protein expression (IRS) with stages and grades (low and high grade) [16]. Yet, in other cancers, we reported similar pattern of SPAG9 expression in breast cancer in early stages and grades [10]. Another example demonstrating such association of high P7 protein expression (>20% cells) in primary breast cancer cells with cancer spread and recurrence has been reported [25]. Likewise, an isoform of cyclin E in stage I breast cancer revealed an association with aggressive phenotype [26]. In addition, expression of KLF4 in early-stage breast intra-ductal carcinoma has also been reported to be associated with aggressive phenotype [27]. It is quite likely that SPAG9 is involved in initial carcinogenesis but acquisition of aggressive phenotype (high grades) may require additional parallel effectors pathways, which needs to be further, investigated. Nevertheless, in context of clinical implications, high SPAG9 expression in early stages and grades may have applications as early diagnostic biomarker for bladder TCC patients.

CT antigens are known to generate spontaneous humoral and cellular immune responses in cancer patients [18]. In the present study, we demonstrated generation of humoral response against SPAG9 in a significant number of bladder TCC patients consistent with the known immunogenicity of this antigen in other cancers [8]–[13]. In an analysis of 125 cases of bladder TCC, 95% patients with *SPAG9* mRNA-positive tumors had generated humoral response against SPAG9: no patients with *SPAG9* mRNA-negative tumors had SPAG9 antibody. However, the remaining 5% bladder TCC patients with SPAG9-expressing tumors did not have detectable SPAG9 antibodies. It is possible that the generation of SPAG9 humoral response depends upon the MHC genetics of the cancer patients classifying them as responders or non-responders to SPAG9 antigenicity. It is important to mention that our findings in majority of superficial non-muscle invasive bladder cancer patients (78%) exhibiting immune response against SPAG9 protein, supports its potential role as a serum biomarker for development of less invasive detection system.

In order to investigate the potential role of SPAG9 in bladder tumorigenesis, we further investigated the SPAG9 expression in various bladder cancer cells of different histotypes. SPAG9 expression was observed in all bladder cancer cell lines of different histological types. The cell surface compartment is of substantial interest in identification of tumor specific proteins for developing therapeutic targets for the cancer treatment. Interestingly, our flow cytometric data analysis revealed that all bladder cancer cells showed cell surface localization of SPAG9. Hence, these findings suggest that SPAG9 protein expression may have important implications in vaccine development by employing active or passive immunization or dendritic cells based approaches for better cancer management programmes and warrants future studies.

Abnormal cellular proliferation, migration and invasion are the important hallmarks of cancer and critical for tumorigenesis and cancer progression. Therefore, we investigated the effect of *SPAG9* knockdown on cellular proliferation, migration and invasion in high grade invasive bladder cancer cell UM-UC-3. We demonstrated that SPAG9 siRNA transfected cells accumulated in G_0_–G_1_ phase as compared with scrambled siRNA transfected cells. Similar to our observations, *PRAME* (another CT antigen) knock-down was reported to cause cell cycle arrest in G1 phase [28]. These observations suggest that *SPAG9* knock-down attenuates cellular proliferation and arrests cell cycle in G_0_–G_1_ phase in bladder cancer cells which is marked by up-regulation of p16 and p21. Consequently, this resulted in down-regulation of various cyclins and CDKs including cyclin E, cyclin D and cyclin B, CDK1 and CDK4 which lead to accumulation of cells in G_0_–G_1_ phase. Migration and invasion of bladder cancer cells remains a major clinical problem due to the lack of effective specific therapies. In the present study, we demonstrated that down-regulation of *SPAG9* was able to inhibit cell migration and invasion of UM-UC-3 cells. The fact that siRNA against *SPAG9* inhibit cancer cellular proliferation, migration and invasion of bladder cancer cells suggests that SPAG9 expression may be involved in early spread and progression of bladder cancer. However, future studies are warranted to validate the findings.

## Conclusion

The present study demonstrated that the majority of bladder TCC patients expressed SPAG9 gene and protein irrespective of various tumor stages and grades. However, SPAG9 expression was more abundant in early stages and low grades. Our studies in UM-UC-3 cells demonstrated the involvement of SPAG9 in cellular proliferation, cell migration and invasion. Therefore, our data showing high SPAG9 expression in early stages and low grades indicates its putative role in tumorigenesis. Further, SPAG9 association with malignant properties acquisition is suggestive of its possible role in acquisition of the invasive phenotype of bladder cancer and warrants further studies. Thus, SPAG9 could be a potential biomarker and therapeutic target for better management of the bladder cancer patients.
